# Targeted expression of BikDD combined with metronomic doxorubicin induces synergistic antitumor effect through Bax activation in hepatocellular carcinoma

**DOI:** 10.18632/oncotarget.4278

**Published:** 2015-06-17

**Authors:** Huei-Yue Dai, Hui-Yu Chen, Wei-Chen Lai, Mien-Chie Hung, Long-Yuan Li

**Affiliations:** ^1^ Center for Molecular Medicine, China Medical University Hospital, Taichung 404, Taiwan; ^2^ Graduate Institute of Cancer Biology, China Medical University, Taichung 404, Taiwan; ^3^ Department of Biotechnology, Asia University, Taichung 413, Taiwan; ^4^ Department of Molecular and Cellular Oncology, The University of Texas MD Anderson Cancer Center, Houston, TX, USA

**Keywords:** synergistic antitumor effect, combination therapy, orthotopic animal model, hepatocellular carcinoma, metronomic chemotherapy

## Abstract

Conventional chemotherapy is commonly used to treat advanced non-resectable hepatocellular carcinoma (HCC) but this treatment modality has not demonstrated convincing survival benefit in HCC patients. Our previous studies indicated that targeted expression of therapeutic BikDD driven by a liver cancer-specific α-fetoprotein promoter/enhancer (eAFP) in the VISA backbone (eAFP-VISA-BikDD) significantly and specifically kills HCC cells in multiple orthotopic animal models. To enhance its therapeutic efficacy, we combined eAFP-VISA-BikDD with chemotherapeutic agents and found that eAFP-VISA-BikDD plus doxorubicin (Dox) or 5-fluorouracil (5-FU) demonstrated synergistic cytotoxicity in HCC cells. Specifically, the combination of eAFP-VISA-BikDD plus Dox markedly induced apoptosis via increased Bax mitochondrial translocation and cytoplasmic cytochrome c release. Compared with either agent alone, a low dose of Dox combined with eAFP-VISA-BikDD induced better antitumor effect and prolonged longer survival of mice in two orthotopic liver cancer xenograft models. Our findings provide strong preclinical support for evaluating the combined therapy of eAFP-VISA-BikDD and Dox in a clinical setting as a treatment option for HCC.

## INTRODUCTION

Hepatocellular carcinoma (HCC) is one of the most common and aggressive malignancies in the world, particularly in Asia and Africa where high prevalence of hepatitis B and C infection has been reported. There are several treatment modalities for early-stage HCC, such as surgical resection, liver transplantation, radiofrequency ablation, and transarterial chemoembolization [1, 2]. Although surgical resection and liver transplantation are considered curative, only a small number of patients are candidates. The poor prognosis of HCC is mainly due to its high propensity for intrahepatic metastasis and tumor recurrence.

Patients with advanced HCC are frequently treated with chemotherapeutic drugs, such as doxorubicin (Dox) and 5-fluorouracil (5-FU) [3–5]. Dox, a topoisomerase II poison, induces apoptosis by disrupting the mitochondrial membrane potential and activating the caspases. 5-FU is an antimetabolite drug that inhibits thymidylate synthase and thereby induces cell death. Unfortunately, there is no convincing survival advantage to using chemotherapeutic agents either as a single agent or in combination to treat advanced HCC [5, 6]. Interestingly, however, the combination of chemotherapy and gene therapy has demonstrated enhanced therapeutic effects and prolonged life span in multiple tumor xenograft models, including those of ovarian, breast, and lung cancers [7–10]. Moreover, results from a phase II clinical trial (NCT00044993) that evaluated the combination of Dox and AdCMV-p53 gene therapy for stage II and III breast cancer patients demonstrated a significant improvement in response rate compared with those receiving chemotherapy alone [11].

HCC is highly refractory to cytotoxic chemotherapy partly due to the increased expression of multidrug-resistance genes, which allow active efflux of chemotherapeutic agents. An imbalance of Bcl-2 family members also plays a role in the chemoresistance of HCC [12]. Functionally, Bcl-2 family members are divided into two major groups, anti-apoptotic and pro-apoptotic. Overexpression of anti-apoptotic Bcl-2 and Bcl-xL proteins rendered HCC cells more resistant to chemotherapeutic agents, such as staurosporine, Dox, and paclitaxel, in human HCC whereas reduced level of Bcl-2 sensitized cancer cells to these agents [13–15]. Expression of Bcl-xL was shown to be higher in HCC than in adjacent normal liver [13], and enhanced Bcl-xL expression in HCC correlated with poorer prognosis [16]. In contrast, ectopic expression of natural born killer (Nbk; also known as Bcl-2 interacting killer or Bik) sensitized breast cancer cells to drug-induced apoptosis and revert acquired chemoresistance [17].

Bik is a BH3-only protein of the Bcl-2 family that promotes apoptosis. Bik-induced cell death is mediated by its interaction with anti-apoptotic proteins, such as Bcl-2, Bcl-xL and Mcl-1, via its BH3 domain, thereby releasing pro-apoptotic proteins Bax and Bak to disrupt the mitochondrial membrane potential, resulting in cytochrome c release and subsequent activation of caspases [18–20]. The activity of Bik is regulated by post-translational modifications [21]. For example, phosphorylation of the Bik at Thr33 and Ser35 increased its apoptotic potency [22]. Mutation of these two phosphorylation sites to create a constitutively phosphorylated Bik by aspartic acid substitutions (BikDD) resulted in higher binding affinity for the antiapoptotic proteins and induced apoptosis more potently than its wild-type counterpart [23].

Overexpression of BikDD has been demonstrated to potently reduce tumor growth in xenograft tumor model of multiple types of cancer [24–26]. Specifically, an eAFP-VISA-BikDD vector was designed to induce targeted expression of BikDD in HCC cells by controlling its expression under a liver cancer-specific α-fetoprotein promoter/enhancer (eAFP), whose activity can further enhanced by the VP16-GAL4-WPRE integrated systemic amplifier (VISA) module [25, 26] to a level similar or comparable to that of the non-specific cytomegalovirus (CMV) promoter. Systemic administration of liposome-encapsulated eAFP-VISA-BikDD effectively repressed tumor growth and prolonged survival in multiple xenograft and syngeneic orthotopic HCC mouse models [26]. In addition to its potent therapeutic efficacy, systemic treatment of a high dose of eAFP-VISA-BikDD/liposome was shown to be safe in animal study [26]. Here, we investigate the potential therapeutic efficacy of eAFP-VISA-BikDD gene therapy in combination with conventional chemotherapeutic agents in HCC cell lines and in orthotopic liver cancer tumor mouse models and explore potential mechanisms by which the combination treatment induces apoptosis.

## RESULTS

### eAFP-VISA-BikDD combined with Dox or 5-FU produces synergistic cytotoxic effects in HCC cells

**Figure 1:**
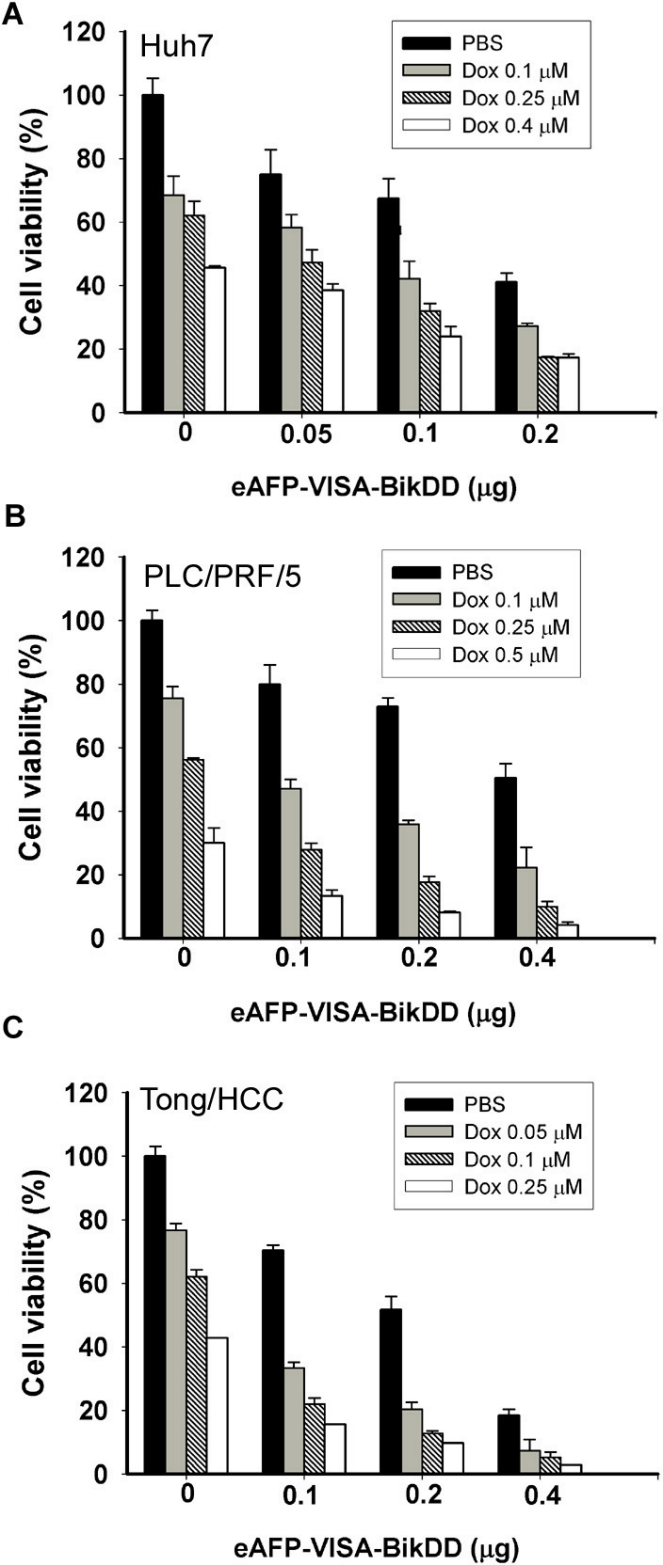
Cytotoxic effects of eAFP-VISA-BikDD plus Dox against human HCC cell lines *in vitro* Huh7 **A.** PLC/PRF/5 **B.** and Tong/HCC **C.** cells were transfected with indicated concentrations of eAFP-VISA-BikDD by using extruding DOTAP:cholesterol liposomes. Transfection mixtures were removed after 4 hours, and fresh DMEM complete medium containing the indicated concentration of Dox was added for an additional 72 hours followed by cytotoxicity analysis by WST-1 assay. Relative cell viability was normalized to untreated cells (set as 100%) and expressed as mean ±SD.

Chemotherapeutic agents, such as Dox and 5-FU, are commonly used to treat advanced non-resectable primary and metastasis HCC. Previously, we demonstrated that non-viral eAFP-VISA-BikDD gene therapy significantly reduced tumor growth in mouse models with a relatively safe profile [26]. Here, we tested a potentially synergistic cytotoxic effect of eAFP-VISA-BikDD plus Dox or 5-FU on HCC cells by WST-1 assay. First, we validated the cytotoxic effect eAFP-VISA-BikDD (by transfection) on HCC cell proliferation. As expected, expression of BikDD effectively reduced cell viability in all three HCC cell lines (Figure 1, black bars). We then treated HCC cells with eAFP-VISA-BikDD plus Dox at various concentrations and found that the combination induced a dose-dependent cytotoxic effect. The IC_50_ (the half maximal inhibitory concentration) of Dox in Huh7 (Figure 1A), PLC/PRF/5 (Figure 1B), and Tong/HCC (Figure 1C) was 0.43, 0.29 and 0.23 μM, respectively. Comparable growth inhibition was observed at approximately 0.1 μM Dox when cells were also transfected with 0.1 μg of eAFP-VISA-BikDD, suggesting that BikDD sensitized HCC cells to Dox-induced cell death.

**Figure 2:**
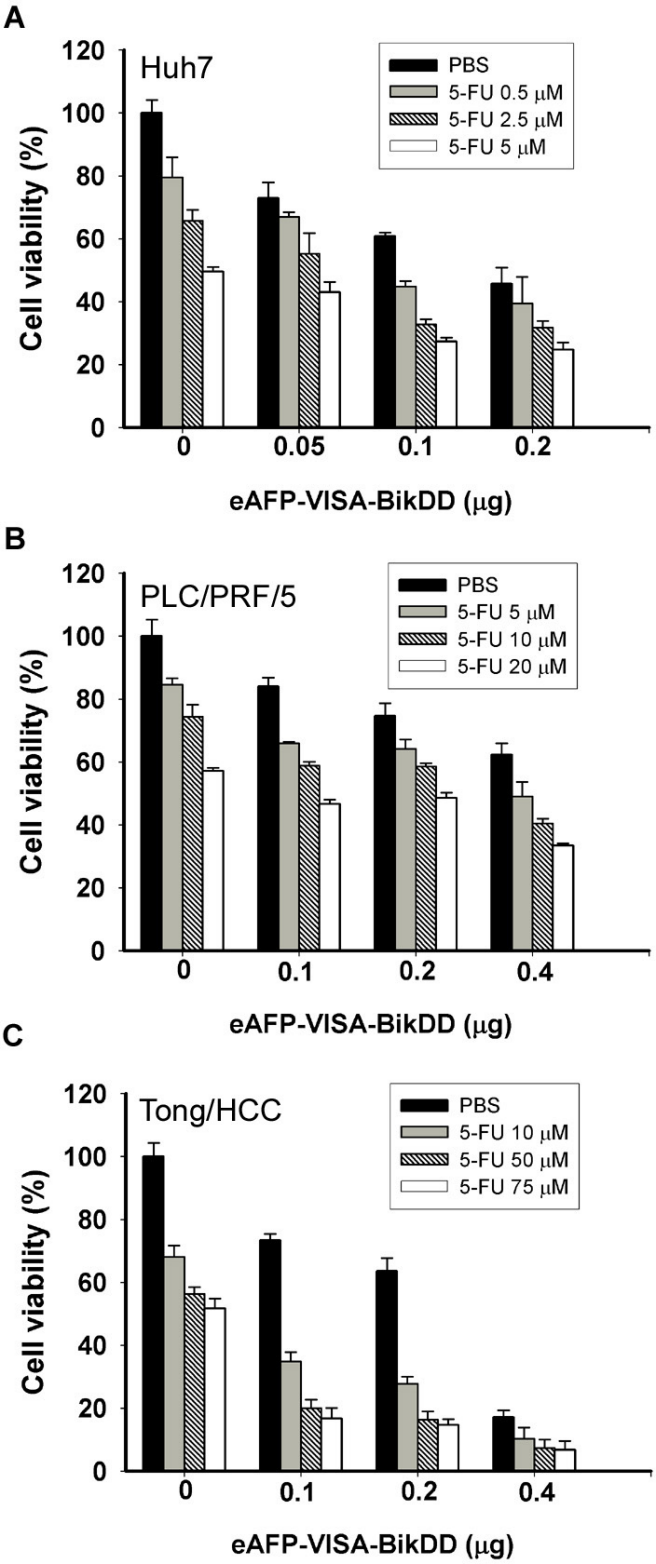
Cytotoxic effects of eAFP-VISA-BikDD plus 5-FU against human HCC cell lines *in vitro* Huh7 **A.** PLC/PRF/5 **B.** and Tong/HCC **C.** cells were transfected for 4 hours with indicated concentrations of eAFP-VISA-BikDD by using extruding DOTAP:cholesterol liposomes. Transfection mixtures were removed after 4 hours, and fresh DMEM complete medium containing the indicated concentration of 5-FU was added for an additional 72 hours followed by cytotoxicity analysis by WST-1 assay. Relative cell viability was normalized to untreated cells (set as 100%) and expressed as mean ±SD.

Next, we calculated the combination index (CI) according to the Chou and Talalay method [27] and plotted against the fraction affected (Fa) for the eAFP-VISA-BikDD and Dox combination in HCC cells (Supplementary Figure S1) and found that it exhibited a synergistic cytotoxic effect (CI values < 1, = 1 and > 1 indicate synergism, additive effect and antagonism, respectively). Similar results were observed when eAFP-VISA-BikDD was combined with 5-FU (Figure 2 and Supplementary Figure S2). Together, our findings suggest that lowering the dose of chemotherapy may be feasible by incorporating eAFP-VISA-BikDD gene therapy to achieve similar cancer cell killing effect at higher doses without the associated adverse side effects.

### eAFP-VISA-BikDD plus Dox is more effective than eAFP-VISA-BikDD plus 5-FU under clinically achievable concentrations

**Figure 3:**
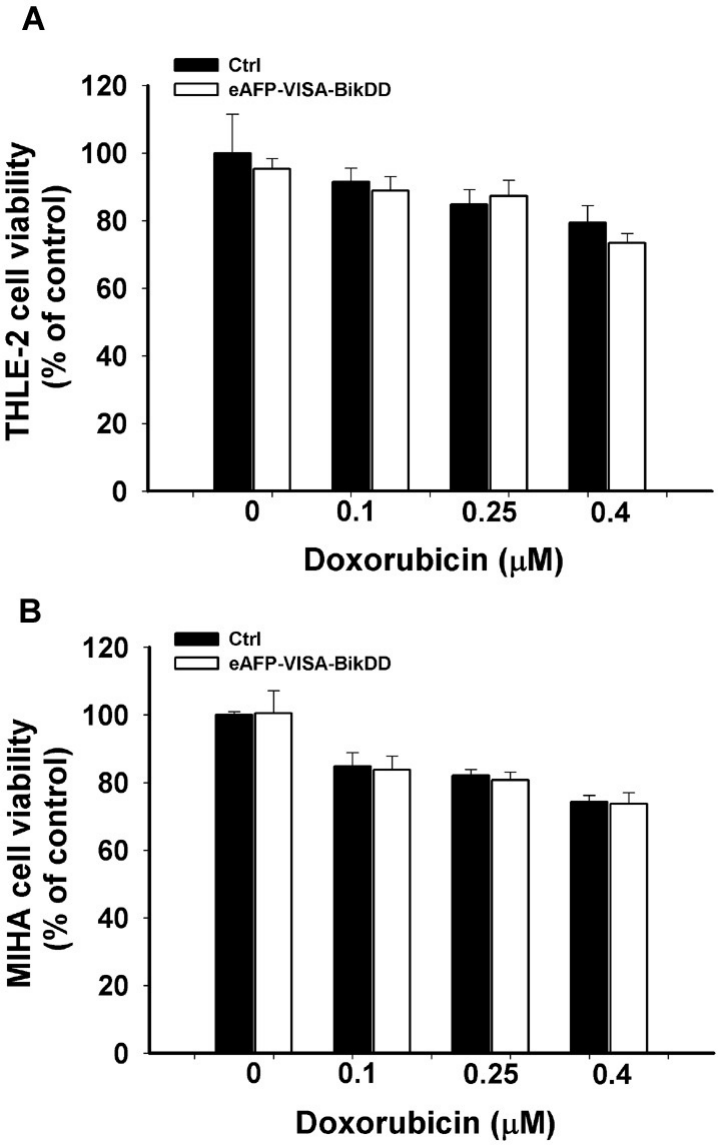
Cytotoxic effects of combining eAFP-VISA-BikDD and Dox in normal liver cell lines THLE-2 **A.** and MIHA **B.** cells were transfected with control (Ctrl) or eAFP-VISA-BikDD for 4 hours. Transfection mixtures were then replaced by fresh complete medium containing the indicated concentration of Dox for an additional 72 hours. At the end of incubation, cytotoxic effects were evaluated by WST-1 assay. Relative cell viability was normalized to untreated cells (set as 100%). Data represent mean ±SD. *N* = 3.

To identify a more clinically relevant combination treatment, we sought to compare the synergistic effect between eAFP-VISA-BikDD/Dox and eAFP-VISA-BikDD/5-FU at clinically relevant concentrations. The steady-state plasma concentration of Dox [28–30] and 5-FU [31–33] in patients ranged from 0.025 to 0.25 μM and 0.192 to 192 μM, respectively, after drug administration. Dose monitoring of 5-FU to achieve maximum clinical efficacy and reduce side effect in patients indicated that a 5-FU plasma concentration between 450 to 550 μg/L (equivalent to 3.46 to 4.23 μM) consistently yielded optimal clinical efficacy and safety in patients [33]. Therefore, we re-analyzed the CI values at higher fraction affected (Fa > 0.5) for eAFP-VISA-BikDD plus clinically relevant dose of Dox (0.1 μM; Supplementary Figure 3A) or 5-FU (5 μM; Supplementary Figure 3B). A CI value between 0.85 − 0.9 indicates slight synergism; 0.7 − 0.85 moderate synergism; 0.3 − 0.7 synergism; 0.1 − 0.3 strong synergism, A higher percentage of data points (CI < 0.7) were observed in HCC cell lines treated with eAFP-VISA-BikDD/Dox (85%) compared with eAFP-VISA-BikDD/5-FU (50%). Notably, strong synergism (CI < 0.3) was only observed in eAFP-VISA-BikDD plus Dox but not 5-FU. These findings indicate that the clinically relevant concentration of Dox can be used with the eAFP-VISA-BikDD gene therapy in combination to effective kill HCC cells. In addition to therapeutic efficacy in cancer cells, we also investigated the effect of eAFP-VISA-BikDD plus Dox on normal liver cell lines with results showing limited damage to normal liver cells *in vitro* (THLE-2 and MIHA; Figure 3A and 3B). On the basis of these findings, we utilized the eAFP-VISA-BikDD/Dox combination for all subsequent experiments.

### eAFP-VISA-BikDD plus Dox increases Bax activation, cytochrome c release, and cleaved caspases-3 expression

**Figure 4:**
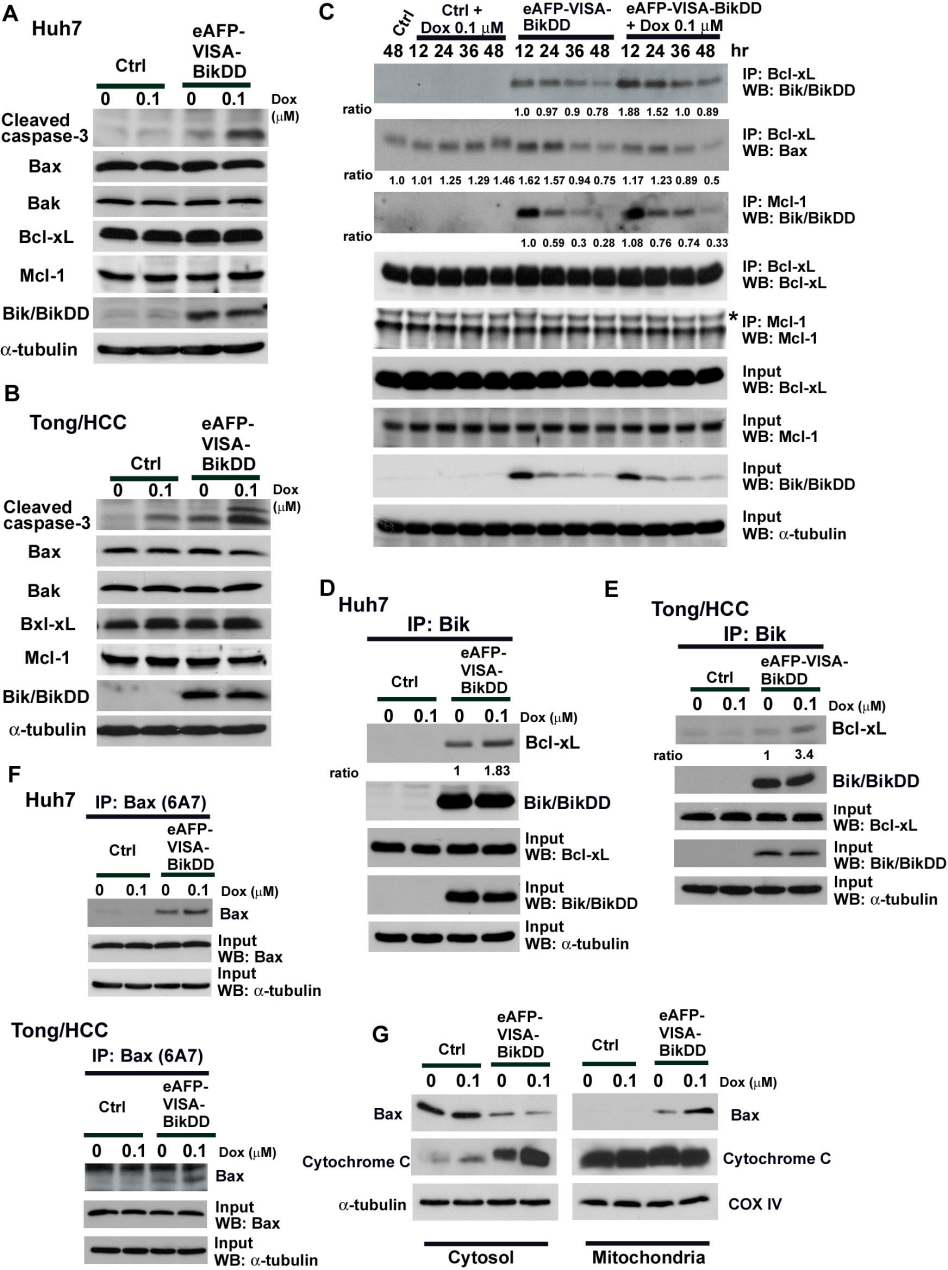
Effects of eAFP-VISA-BikDD combined with DOX on apoptosis-related protein expression in HCC cell lines Huh7 **A.** and Tong/HCC **B.** cells were transfected with control (Ctrl) vector or eAFP-VISA-BikDD. After 4 hours of incubation, transfection mixtures were replaced by fresh DMEM complete medium or DMEM complete medium containing Dox (0.1 μM) for 72 hours. Cell lysates were collected for Western blot analysis. **C.** Huh7 cells were transfected for 4 hours with vector control (Ctrl) or eAFP-VISA-BikDD. After incubation, the transfection mixture was removed then replaced with DMEM complete medium or DMEM complete medium containing Dox (0.1 μM) for additional 12–48 hours. Cells were harvested at the indicated time points and subjected to immunoprecipitation with anti-Bcl-xL and anti-Mcl-1 antibodies. **D, E.** Cells were treated as described above and lysates were harvested at 24 hours after Dox administration and subjected to immunoprecipitation by anti-Bik antibody in 1% CHAPS lysis buffer. Equal amounts of proteins from different treatment were separated by SDS-PAGE and then incubated with anti-BiK antibody. The anti-Bik polyclonal antibody recognizes both wild-type and mutant form Bik. **F.** Supernatant was harvested from precipitated immune complexes from (D) and (E) and subjected to secondary immunoprecipitation with anti-Bax (6A7) antibody. **G.** Bax mitochondrial translocation and cytochrome c release in combination therapy. Huh7 cells were transfected with control vector or eAFP-VISA-BikDD for 4 hours and then incubated with DMEM complete medium or DMEM complete medium containing Dox for additional 36 hours. Cell lysates were harvested and subjected to subcellular fractionation experiments. Western blot analysis was performed to determine the levels of Bax and cytochrome c. Antibody to cytochrome c oxidase IV antibody (COX IV) was used as mitochondrial loading control.

To determine whether the synergistic growth inhibition by eAFP-VISA-BikDD/Dox is attributed to enhanced apoptosis, we analyzed cell viability by propidium iodide staining and flow cytometry 72 hours after combination treatment. The percentage of sub-G1 cells was significantly increased after treatment with eAFP-VISA-BikDD, Dox, or the combination compared with the control vector-transfected group (*p* < 0.001, Supplementary Table S1). The level of apoptosis was highest in cells treated with eAFP-VISA-BikDD/Dox in 3 HCC cell lines (*p* < 0.05). Moreover, cells treated with eAFP-VISA-BikDD/Dox had higher expression of cleaved caspase-3 than those treated with each agent alone (Figure 4A and 4B). The expression levels of both proapoptotic (Bax and Bak) and antiapoptotic (Bcl-xL and Mcl-1) did not change upon various treatments (Figure 4A and 4B). In addition, the expression levels of BikDD were similar between cells treated with eAFP-VISA-BikDD alone or eAFP-VISA-BikDD/Dox, suggesting that altered expression of these Bcl2 family proteins may not be involved in the increased apoptosis by the combination treatment. Thus, we next sought to investigate the molecular mechanisms underlying eAFP-VISA-BikDD/Dox-enhanced apoptosis.

Induction of apoptosis by the Bik/Nbk is mediated by the dissociation of anti-apoptotic proteins (e.g., Bcl-2, Bcl-xL, and Mcl-1) from pro-apoptotic proteins (e.g., Bax or Bak) to execute cell death signals [18, 34]. Previous studies have indicated that Huh-7 cells express Bcl-xL at high levels but not Bcl-2 [35]. We screened the protein expression of Bcl-2, Bcl-xL, and Mcl-1 in Huh7, PLC/PRF/5 and Tong/HCC cells and also found that Bcl-xL and Mcl-1 were highly expressed in Huh7, PLC/PRF/5 and Tong/HCC whereas Bcl-2 was barely undetectable (Supplementary Figure S4). We hypothesized that eAFP-VISA-BikDD/Dox may increase apoptosis by enhancing the binding affinity of BikDD to the anti-apoptotic proteins. Huh7 cells were treated with eAFP-VISA-BikDD/Dox followed by immunoprecipitation using anti-Bcl-xL or anti-Mcl-1 antibodies. eAFP-VISA, which does not express BikDD, was used as control for comparison. The results revealed that the interaction between BikDD and Bcl-xL was substantially enhanced in eAFP-VISA-BikDD/Dox-treated cells that peaked at 12–24 hour, which then declined but remained higher than that observed in eAFP-VISA-BikDD-treated cells at 48 hours (Figure 4C). In contrast, cells treated with eAFP-VISA-BikDD alone did not exhibit such increase in BikDD/Bcl-xL interaction (Figure 4C). The interaction between BikDD and Mcl-1 was initially similar between eAFP-VISA-BikDD and eAFP-VISA-BikDD/Dox-treated cells (12∼24 hours after Dox treatment). However, persistent binding of BikDD to Mcl-1 was observed from a longer incubation period (about 36 hours) in eAFP-VISA-BikDD/Dox-treated but not eAFP-VISA-BikDD cells. BikDD expression in input lysates was reduced over time in eAFP-VISA-BikDD and eAFP-VISA-BikDD/Dox-treated cells due to transient transfection. Expression of endogenous Bik protein did not unchanged, and interaction between Bik and Bcl-xL or Mcl-1 was not detected even after 48 hours of Dox treatment. We further validated the interaction between BikDD and anti-apoptotic Bcl-2 family proteins by reciprocal immunoprecipitation using antibody against Bik (Figure 4D) which showed that the binding of BikDD to Bcl-xL was increased when we added Dox to the eAFP-VISA-BikDD gene therapy. Similar results were observed in Tong/HCC cells (Figure 4E). Taken together, these results indicate Dox prolongs the binding of BikDD to Bcl-xL or Mcl-1, leading to enhance apoptosis.

On the basis of these observations, we asked whether higher levels of activated Bax or Bak are released as a result of enhanced binding of BikDD to Bcl-xL or Mcl-1 to promote cell death. To this end, we collected supernatant from first immunoprecipitation with anti-Bik followed by a second immunoprecipitation with anti-Bax (6A7) or anti-Bak. The Bax (6A7) and Bak antibodies used in the current study specifically recognize the epitope at the N-terminus of activated Bax and Bak. We observed higher levels of activated Bax in cells treated with eAFP-VISA-BikDD/Dox than eAFP-VISA-BikDD or Dox alone in Huh7 and Tong/HCC cells (Figure 4F). However, the levels of activated Bak in combination therapy were similar to that in eAFP-VISA-BikDD gene therapy alone (Supplementary Figure S5). Results from subcellular fractionation also revealed higher levels of Bax in the mitochondria and cytochrome c released into cytosol in cells treated with eAFP-VISA-BikDD/Dox (Figure 4G). These findings indicate that combination therapy promotes apoptosis likely by increasing the binding of BikDD to anti-apoptotic proteins to release and activate Bax.

### Synergistic anticancer effect of eAFP-VISA-BikDD/Dox in orthotopic xenograft mouse model

**Figure 5:**
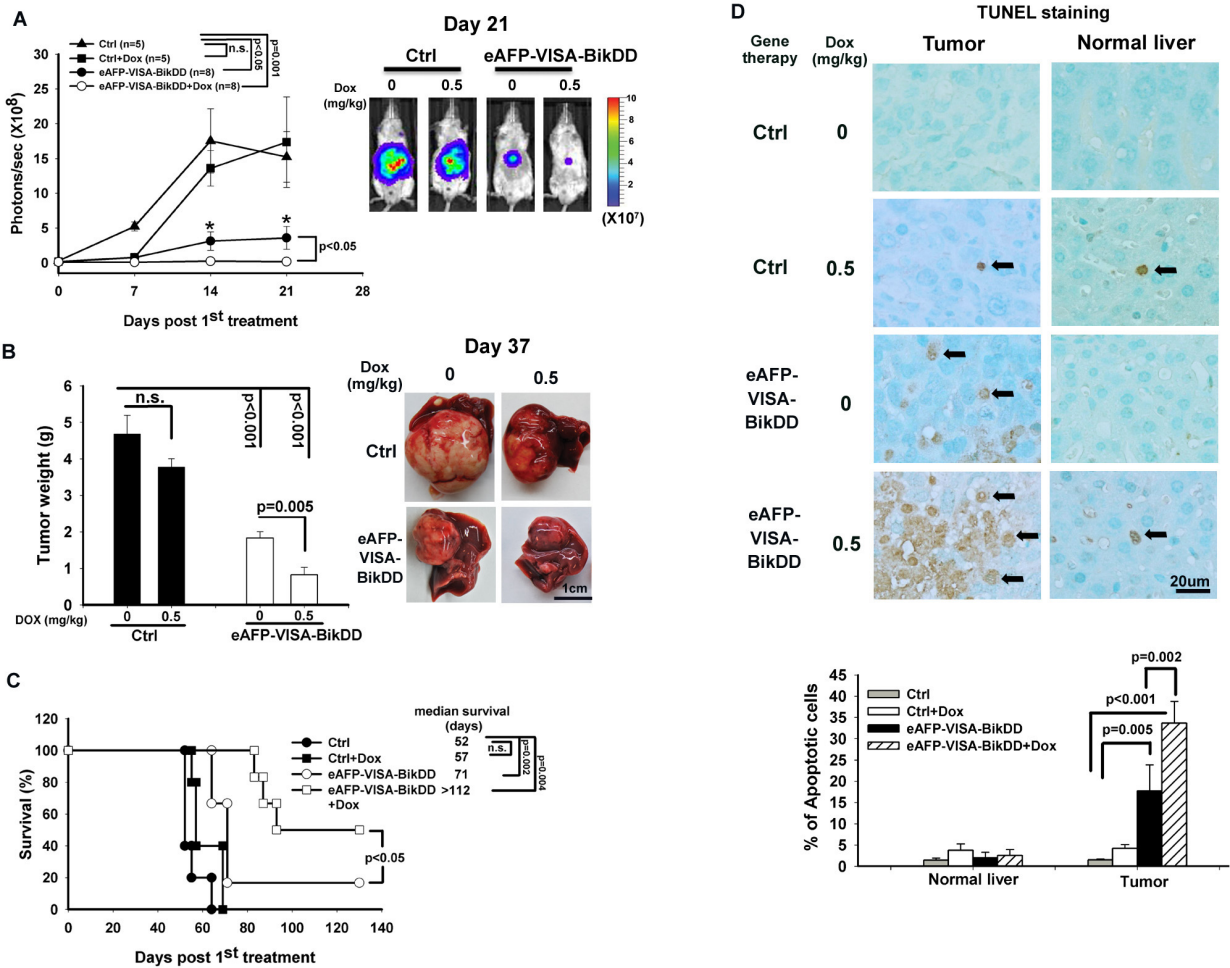
Antitumor effect of eAFP-VISA-BikDD combined with Dox in an orthotopic xenograft mouse model **A.** Huh7 cells were orthotopically inoculated into the left liver lobe of SCID mice. One week after tumor inoculation, mice were randomly divided into four groups for treatment. Mice received systemic intravenous injection of control vector or eAFP-VISA-BikDD encapsulated in liposomes twice a week for four weeks. Dox (0.5 mg/kg) or normal saline was administered once a week by tail vein injection for four weeks. Tumor growth was monitored weekly by *in vivo* imaging system. **B.** Thirty-seven days after initial treatment, mice were sacrificed and liver tumors were dissected and weighed. Data represent mean ±SEM. *N* = 5. Representative images of tumor tissues from mice after the indicated treatment are shown on the right. **C.** Survival rate of mice after the indicated treatment. n.s., not significant. **D.** Detection of apoptosis in liver tissues by TUNEL assays. Tissues collected from (A) were fixed with 10% neutral buffered formalin, and TUNEL-positive cells were stained and counted from four randomly selected fields per section. Arrows indicate TUNEL-positive apoptotic cells. Quantitation is shown below. Data represent mean ±SD.

To evaluate the potential therapeutic efficacy of eAFP-VISA-BikDD/Dox in liver cancer, we established an orthotopic xenograft mouse model of hepatocellular carcinoma. We first determined the dosage and frequency of Dox administration for this study. The conventional-dose schedule for Dox in humans is 60 to 75 mg per m^2^ every 21 to 28 days. It is well known that high doses of Dox induce adverse side effects, such as heart failure. To reduce toxicities, metronomic chemotherapy (chronic low doses of chemotherapeutic drugs) is another option for drug administration. Several studies have reported that weekly low-dose Dox produces equivalent antitumor effects compared with conventional high-dose intermittent Dox [36, 37]. Therefore, we examined the efficacy of weekly low-dose Dox in combination with eAFP-VISA-BikDD twice a week in a mouse tumor model of HCC. To this end, severe-combined immune deficient (SCID) mice bearing the orthotopic Huh7-Luc cells were systemically administered eAFP-VISA-BikDD alone or in combination with low-dose Dox. Tumor growth was monitored weekly by non-invasive *in vivo* bioluminescence imaging system. Mice treated with eAFP-VISA-BikDD alone or combined with Dox (0.5 mg/kg) had significantly smaller tumors compared with those treated with vector control at day 21 after initial treatment (Figure 5A; Representative images from IVIS shown on right). Moreover, mice treated with eAFP-VISA-BikDD/Dox had more statistically significant reduction in tumor growth than those treated with eAFP-VISA-BikDD alone (*p* < 0.05) or vector control (*p* < 0.001) at day 14 and day 21 after the initial treatment. Similar results were also obtained when we measured the tumor weight at 37 days after initial treatment (Figure 5B). These results are consistent with our previous studies that demonstrated similar tumor growth inhibition by eAFP-VISA-BikDD [26]. Weekly treatment of low-dose Dox (0.5 mg/kg) for 4 weeks did not affect tumor weight compared with the control group. Strikingly, the combination of eAFP-VISA-BikDD and Dox (0.5 mg/kg) had better antitumor efficacy than eAFP-VISA-BikDD monotherapy (*p* = 0.005). The average tumor weight was 1.834 ± 0.17g and 0.825 ± 0.2 g for eAFP-VISA-BikDD and eAFP-VISA-BikDD/Dox-treated groups, respectively. eAFP-VISA-BikDD/Dox also prolonged mice survival compared to eAFP-VISA-BikDD (Figure 5C; median survival: combined therapy, > 112 days vs. eAFP-VISA-BikDD, 71 days; *p* < 0.05). In addition, TUNEL staining of mice tumor tissues indicated the percentage of apoptotic cells was highest in eAFP-VISA-BikDD/Dox-treated group followed by eAFP-VISA-BikDD alone, Dox alone, and control in that order (Figure 5D; quantitation shown below). The results from TUNEL assay were consistent with those from tumor growth inhibition and survival analysis showing that eAFP-VISA-BikDD/Dox effectively induces apoptosis. Importantly, eAFP-VISA-BikDD/Dox as well as low-dose Dox treatment had very little effect on normal liver cells and did not significantly reduce mice body weight compared with initial body weight before treatment (Supplementary Figure S6). Hence, this combination regimen may provide a relatively safe and effective therapeutic option for the treatment of hepatocellular carcinoma as demonstrated in the animal model.

### eAFP-VISA-BikDD/Dox inhibits tumor growth and metastasis and prolongs survival time in orthotopic syngeneic mouse model

**Figure 6:**
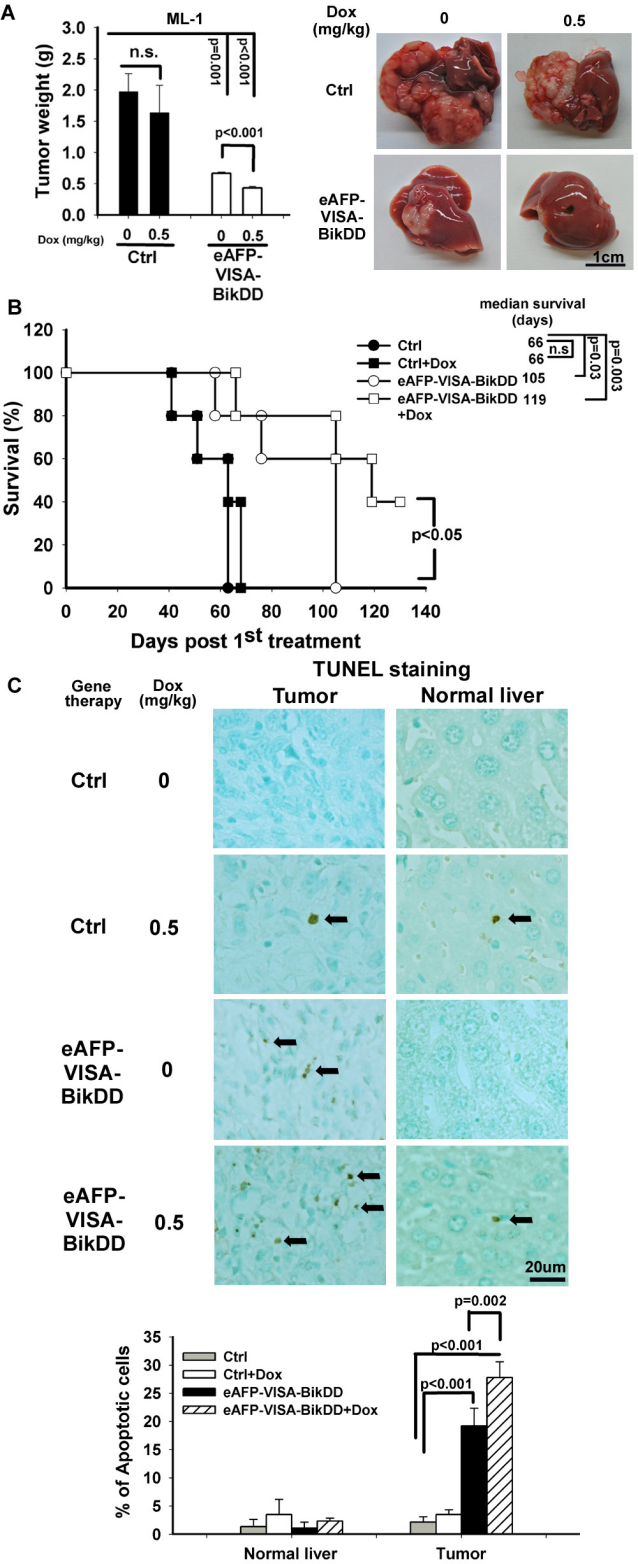
Antitumor effect of eAFP-VISA-BikDD combined with Dox in an orthotopic syngeneic mouse model **A.** Left, ML-1 cells were orthotopically inoculated into the left liver lobe of BALB/c mice. One week after tumor inoculation, mice were randomly divided into four groups for treatment. Mice received systemic intravenous injection of control vector or eAFP-VISA-BikDD encapsulated in liposomes twice a week for four weeks. Dox (0.5 mg/kg) or normal saline was administered once a week by tail vein injection for four weeks. Thirty-seven days after initial treatment, mice were sacrificed and liver tumors were dissected and weighed. Data represent mean ±SEM. *N* = 5. Representative images of tumor tissues from mice after the indicated treatment are shown on the right. **B.** Survival rate of mice after the indicated treatment. n.s., not significant. **C.** Detection of apoptosis in liver tissues by TUNEL assays. Tissues collected from (A) were fixed with 10% neutral buffered formalin, and TUNEL-positive cells were stained and counted from four randomly selected fields per section. Arrows indicate TUNEL-positive apoptotic cells. Quantitation is shown below. Data represent mean ±SD.

To evaluate the combination therapy in a syngeneic animal model with normal host immune response, we first evaluated the cytotoxic effect of eAFP-VISA-BikDD/Dox *in vitro* in ML-1 mouse HCC cells by calculating the CI value. As shown in the Fa-CI plot (Supplementary Figure S7), eAFP-VISA-BikDD/Dox exhibited synergistic cytotoxic effect, with CI values below 1 indicating synergism. We then examined the efficacy of this combination in mice with ML-1 tumors. Mice treated with eAFP-VISA-BikDD/Dox had significantly smaller tumors and longer survival rate compared with the control group (Figure 6A and 6B). The number of TUNEL-positive cells in the tumors of eAFP-VISA-BikDD/Dox-treated group was 1.4- and 13-fold higher than in those of the eAFP-VISA-BikDD-treated and control group, respectively (Figure 6C; quantitation shown below). Remarkably, mice that received systemic administration of eAFP-VISA-BikDD gene therapy alone or combined Dox had less pulmonary nodules compared with those that received no treatment (Supplementary Table S2), suggesting that eAFP-VISA-BikDD may also suppress metastasis.

## DISCUSSION

HCC is highly malignant and resistant to chemotherapeutic agents and has high propensity of intrahepatic and extrahepatic metastases. To improve the overall therapeutic efficacy, especially in treating unresectable HCC, combination therapies are being explored to maximize therapeutic advantages while reducing potential side effects and complications. Among various combination regimens, studies on gene therapy and chemotherapy are of particular interest [9, 10]. Here, we demonstrated that the combination of eAFP-VISA-BikDD gene therapy and clinically achievable concentration of Dox is both effective and safe in the animal models tested.

Compared with conventional high-dose chemotherapy, metronomic chemotherapy, which is administered at low doses and more frequent intervals, has more clinical benefits not only in the reduction of adverse drugs toxicity but also in targeting both proliferating tumor cells and tumor-associated endothelial cells [36, 38]. This approach has become widely utilized by oncologists in clinical trials to treat a variety of human cancers [39–42]. Generally, conventional Dox is administrated in mice at a dose of 10 mg/kg every 3 weeks. In this study, while metronomic Dox (0.5 mg/kg, once per week for consecutive 4 weeks) did not exhibit therapeutic efficacy in orthotopic HCC animal models, when combined with eAFP-VISA-BikDD, significant tumor regression, prolonged survival, and even inhibition of distant metastasis were observed (Figures 5 and 6 and Supplementary Table S2). The effect of eAFP-VISA-BikDD/Dox on normal liver tissues was negligible (Figure 5D and 6C), supporting that this combination regimen not only provides therapeutic efficacy but also retains safety in orthotopic HCC mice models.

The mechanisms of Dox-induced apoptosis in normal and tumor cells are different [43]. Wang *et al*. showed that Dox induces apoptosis through enhanced p53 expression, leading to caspase 3 activation. A similar phenomenon was also observed when Dox was combined with p53 gene therapy in the treatment of HCC [44]. We did not detect wild-type p53 expression in HCC cell lines after Dox treatment because all the HCC cell lines (Huh7, Tong/HCC and PLC/PRF/5) that we used in this study harbor mutant p53. Several studies have also reported enhanced Bik expression in the presence of Dox in various types of human cancers, such as melanoma, lymphoma, and breast cancer [45–47]. However, the levels of endogenous Bik in HCC cells remained undetectable by Western blot analysis even with the addition of Dox. Although our findings did not support the results from the prior studies, the variation in Bik protein expression in response to Dox is likely cell-type dependent. Our study demonstrated that Dox plays a distinctly different role in promoting BikDD-induced apoptosis by enhancing the binding affinity of BikDD to anti-apoptotic proteins although further studies will be required to delineate the mechanism.

In summary, we demonstrated that the eAFP-VISA-BikDD/Dox combination triggers mitochondria-dependent cell apoptosis involving the activation of Bax and caspases-3. Moreover, eAFP-VISA-BikDD sensitizes HCC tumors to Dox treatment. Our findings suggest that the eAFP-VISA-BikDD/Dox combination has great potential as a treatment modality for advanced HCC.

## MATERIALS AND METHODS

### Cell lines and reagents

HCC cell lines Huh7, PLC/PRF/5, and Tong/HCC, were cultured in Dulbecco's modified Eagles's medium (DMEM) supplemented with 10% FBS (Gibco BRL, Rockville, MD) at 37°C with 5% CO_2_. THLE-2, an immortalized normal liver cell line was provided by Dr. Ya-Wen Chen (National Health Research Institutes, Taiwan). The non-tumorigenic immortalized liver cell line MIHA was kindly provided by Dr. Chowdhury (Albert Einstein College of Medicine, New York). Doxorubicin (Dox) and 5-fluorouracil (5-FU) were purchased from Sigma Chemical Co. (St. Louis, MO).

### Transfection and drug administration

Construction and purification of the pUK-eAFP-VISA-BikDD expression plasmid was described previously [26]. HCC cells seeded on 96-well plates at a density of 5, 000 cells per well and then transfected with pUK-eAFP-VISA-BikDD with the indicated plasmid concentration by using extruding DOTAP:cholesterol liposomes. Four hours after incubation, transfection mixture was aspirated and then replaced by fresh complete medium containing various concentrations of chemotherapeutic drugs for an additional 72 hours.

### Cell viability assay

WST-1 assay was performed to assess the killing effect (cytotoxicity) of BikDD, chemotherapeutic agents, or their combination according to the manufacturer's protocol (Roche Diagnostics, Mannhein, Germany). At harvest, ten microliters of WST-1 reagent was added to each well and incubated at 37°C for 3 hours. Absorbance was taken at 450 nm and at 630 nm for the test and background wavelength, respectively, by an ELISA reader. Cell viability was expressed as the percentage of absorbance of the treated cells relative to that of the untreated control cells. The effect of the combination was determined by calculating the combination index (CI) in Calcusyn software using non-constant ratio combination (Biosoft, Ferguson, MO). CI values > 1, = 1 and < 1 represent antagonism, additive effect and synergism, respectively. Fraction affected (Fa) value indicates the fraction of cell death after drug exposure such that *a* value of 0.5 represents 50% of cell killing. The molar ratios of eAFP-VISA-BikDD to Dox in HCC cells are shown in Supplementary Table S3.

### Flow cytometric analysis of DNA content

To detect DNA contents, cells were fixed in ice-cold 70% ethanol at 4°C overnight and then washed with ice-cold PBS twice prior to incubation with freshly prepared propidium iodide (PI) staining solution containing 100 μg/ml RNaseA and 50 μg/ml PI at room temperature for 30 min. At the end of the incubation period, cells were analyzed by flow cytometry.

### Immunoprecipitation and Western blot analysis

Cells were lysed in 1% CHAPS lysis buffer containing proteinase inhibitors for 10 min on ice. Cell lysate was centrifuged at 12, 000 rpm for 10 min at 4°C to collect supernatant for protein quantification. For immunoprecipitation, an equivalent amount of protein (500 μg) was incubated with anti-Nbk/Bik antibody (Santa Cruz Biotechnology), anti-Bcl-xL (Cell Signaling Technology) or anti-Mcl-1 (BD Pharmingen) overnight at 4°C. After incubation with protein A/G-Sepharose beads on a rotating platform for 3 h, immune complexes were washed with lysis buffer four times and then resuspended in SDS-PAGE sample buffer. Finally, the samples were denatured at 95∼100°C for 5min and then separated by SDS-PAGE. To detect the active form of Bax and Bak, the supernatants were collected from 1^st^ immunoprecipitation with Bik antibody and then incubated with 1 μg of anti-Bax (6A7; BD Pharmingen) or anti-Bak (AM03; Calbiochem) antibody in 300 μl of CHAPS lysis buffer at 4°C overnight. For Western blot analysis, an equal amount of proteins (30 μg) from different lysates were separated on 12∼15% SDS-PAGE and then electroblotted onto PVDF membrane. The blot was probed with the following primary antibodies: anti-Bik (Santa Cruz Biotechnology), anti-Mcl-1 (BD Pharmingen), anti-Bcl-xL (Cell Signaling Technology), anti-Bax (Santa Cruz), anti-Bak (Calbiochem), anti-cleaved caspase-3 (Cell Signaling Technology) and α-tubulin (AC-74, Sigma-Aldrich) followed by horseradish peroxidase-conjugated secondary antibodies. Active (cleaved) caspase-3, a maker for apoptosis, consists of a large (17 kDa) and small (12 kDa) subunit derived from the inactive proenzyme. The cleaved caspase-3 (Asp175) antibody used in this study specifically recognizes the large fragment of activated capsase-3 but not full-length caspase-3 or other cleaved caspases. The anti-Bik polyclonal antibody recognizes both wild-type and mutant Bik. All membranes were detected by the ECL chemiluminescence reaction (GE Healthcare Life Sciences).

### Subcellular fractionation

Cells were harvested at indicated time points and lysed in mitochondria lysis buffer with a Dounce homogenizer until about 90% cells were broken. The homogenate was centrifuged at 1, 000 *g* to pellet the nuclei and unbroken cells. The supernatant was collected and then subjected to centrifugation at 10, 000 *g* for 30 min at 4°C to pellet mitochondria-enriched heavy membrane fraction. The final supernatant was further centrifuged at 100, 000 *g* for 60 min at 4°C to obtain cytosolic fraction.

### Combination treatment in orthotopic animal models

Five-week-old male severe-combined immune deficient (SCID) or BALB/c mice were purchased from BioLASCO Company (Yi Lan, Taiwan, ROC) and were housed in a specific pathogen free condition at China Medical University Laboratory Animal Center. Food and water were available *ad libitum*. All animal experiments were approved by the Laboratory Animal Care and Use Committee of the China Medical University. After anesthesia, mice underwent median laparotomy. Huh7 (5 × 10^5^) or ML-1 cell (10^5^) were inoculated into the subcapsular parenchyma of the left liver lobe by an insulin syringe (Terumo, Elkton, MD). One week after inoculation, mice were randomly assigned to 4 groups. To evaluate the combination therapy, mice were administered DNA/liposome complex (20 μg) twice per week and then Dox (0.5 mg/kg) once per week by tail vein injection for a total of 4 weeks. Tumor growth was monitored weekly by in vivo Imaging System (Xenogen). Thirty-seven days after the initial treatment, all mice were sacrificed and liver tumors dissected and weighed.

### Terminal deoxynucleotidyl transferase dUTP nick end labeling (TUNEL) assay

To investigate cell apoptosis induced by various treatments, an *in situ* TUNEL assay (BioVision, Mountain View, CA) was performed according to the manufacturer's instructions with some modification. Briefly, after deparaffinization and rehydration, tissue sections were permeabilized by proteinase K at RT for 15 min. The slides were washed by PBS twice then immersed in 3% H_2_O_2_/methanol for 5 min to quench the endogenous peroxidase activity. After completion of TdT-end labeling, tissue sections were placed in 2X SSC for 15 min at room temperature to terminate the TdT enzyme reactions and washed twice in PBS. The sections were sequentially incubated with antibody solution and conjugate. Finally, the slides were developed in 3, 3′ diaminobenzidine (DAB) chromogen and counterstained with methyl green. The mean of TUNEL-positive cells was calculated by counting cells from four random fields for each sample.

### Statistical analyses

All data were representative of at least three independent experiments. All *P* values were analyzed by student's *t*-test except for calculation of survival time. Survival curves were calculated by Kaplan-Meier method and compared using the log-rank test (SPSS software). A *P* value < 0.05 was considered statistically significant.

## SUPPLEMENTARY FIGURES AND TABLES



## References

[1] Schwartz M, Roayaie S, Konstadoulakis M (2007). Strategies for the management of hepatocellular carcinoma. Nature clinical practice Oncology.

[2] El-Serag HB, Marrero JA, Rudolph L, Reddy KR (2008). Diagnosis and treatment of hepatocellular carcinoma. Gastroenterology.

[3] Han KH, Park JY (2008). Chemotherapy for advanced hepatocellular carcinoma. Journal of gastroenterology and hepatology.

[4] Tanikawa K (2001). Chemotherapy for highly advanced hepatocellular carcinoma. Journal of gastroenterology and hepatology.

[5] Brown KS (2006). Chemotherapy and other systemic therapies for hepatocellular carcinoma and liver metastases. Seminars in Interventional Radiology.

[6] Topp ZZ, Sigal DS (2013). Beyond chemotherapy: systemic treatment options for hepatocellular carcinoma. Translational Cancer Research.

[7] Sopo M, Anttila M, Sallinen H, Tuppurainen L, Laurema A, Laidinen S, Hamalainen K, Tuunanen P, Koponen JK, Kosma VM, Heinonen S, Alitalo K, Yla-Herttuala S (2012). Antiangiogenic gene therapy with soluble VEGF-receptors -1, -2 and -3 together with paclitaxel prolongs survival of mice with human ovarian carcinoma. International journal of cancer Journal international du cancer.

[8] Lin T, Zhang L, Davis J, Gu J, Nishizaki M, Ji L, Roth JA, Xiong M, Fang B (2003). Combination of TRAIL gene therapy and chemotherapy enhances antitumor and antimetastasis effects in chemosensitive and chemoresistant breast cancers. Molecular therapy : the journal of the American Society of Gene Therapy.

[9] Li D, Zhang Y, Xie Y, Xiang J, Zhu Y, Yang J (2013). Enhanced tumor suppression by adenoviral PTEN gene therapy combined with cisplatin chemotherapy in small-cell lung cancer. Cancer gene therapy.

[10] Zhang M, Garbuzenko OB, Reuhl KR, Rodriguez-Rodriguez L, Minko T (2012). Two-in-one: combined targeted chemo and gene therapy for tumor suppression and prevention of metastases. Nanomedicine (Lond).

[11] Cristofanilli M, Krishnamurthy S, Guerra L, Broglio K, Arun B, Booser DJ, Menander K, Van Wart Hood J, Valero V, Hortobagyi GN (2006). A nonreplicating adenoviral vector that contains the wild-type p53 transgene combined with chemotherapy for primary breast cancer: safety, efficacy, and biologic activity of a novel gene-therapy approach. Cancer.

[12] Fernandez-Luna JL (2008). Regulation of pro-apoptotic BH3-only proteins and its contribution to cancer progression and chemoresistance. Cellular signalling.

[13] Takehara T, Liu X, Fujimoto J, Friedman SL, Takahashi H (2001). Expression and role of Bcl-xL in human hepatocellular carcinomas. Hepatology.

[14] Chun E, Lee KY (2004). Bcl-2 and Bcl-xL are important for the induction of paclitaxel resistance in human hepatocellular carcinoma cells. Biochemical and biophysical research communications.

[15] Luo D, Cheng SC, Xie H, Xie Y (1999). Chemosensitivity of human hepatocellular carcinoma cell line QGY-7703 is related to bcl-2 protein levels. Tumour biology : the journal of the International Society for Oncodevelopmental Biology and Medicine.

[16] Watanabe J, Kushihata F, Honda K, Sugita A, Tateishi N, Mominoki K, Matsuda S, Kobayashi N (2004). Prognostic significance of Bcl-xL in human hepatocellular carcinoma. Surgery.

[17] Radetzki S, Kohne CH, von Haefen C, Gillissen B, Sturm I, Dorken B, Daniel PT (2002). The apoptosis promoting Bcl-2 homologues Bak and Nbk/Bik overcome drug resistance in Mdr-1-negative and Mdr-1-overexpressing breast cancer cell lines. Oncogene.

[18] Gillissen B, Essmann F, Graupner V, Starck L, Radetzki S, Dorken B, Schulze-Osthoff K, Daniel PT (2003). Induction of cell death by the BH3-only Bcl-2 homolog Nbk/Bik is mediated by an entirely Bax-dependent mitochondrial pathway. The EMBO journal.

[19] Chinnadurai G, Vijayalingam S, Rashmi R (2008). BIK, the founding member of the BH3-only family proteins: mechanisms of cell death and role in cancer and pathogenic processes. Oncogene.

[20] Gillissen B, Essmann F, Hemmati PG, Richter A, Richter A, Oztop I, Chinnadurai G, Dorken B, Daniel PT (2007). Mcl-1 determines the Bax dependency of Nbk/Bik-induced apoptosis. The Journal of cell biology.

[21] Jiao S, Wu M, Ye F, Tang H, Xie X (2014). BikDDA, a mutant of Bik with longer half-life expression protein, can be a novel therapeutic gene for triple-negative breast cancer. PloS one.

[22] Verma S, Zhao LJ, Chinnadurai G (2001). Phosphorylation of the pro-apoptotic protein BIK: mapping of phosphorylation sites and effect on apoptosis. The Journal of biological chemistry.

[23] Li YM, Wen Y, Zhou BP, Kuo HP, Ding Q, Hung MC (2003). Enhancement of Bik antitumor effect by Bik mutants. Cancer research.

[24] Sher YP, Tzeng TF, Kan SF, Hsu J, Xie X, Han Z, Lin WC, Li LY, Hung MC (2009). Cancer targeted gene therapy of BikDD inhibits orthotopic lung cancer growth and improves long-term survival. Oncogene.

[25] Xie X, Xia W, Li Z, Kuo HP, Liu Y, Li Z, Ding Q, Zhang S, Spohn B, Yang Y, Wei Y, Lang JY, Evans DB, Chiao PJ, Abbruzzese JL, Hung MC (2007). Targeted expression of BikDD eradicates pancreatic tumors in noninvasive imaging models. Cancer cell.

[26] Li LY, Dai HY, Yeh FL, Kan SF, Lang J, Hsu JL, Jeng LB, Chen YH, Sher YP, Lin WC, Hung MC (2011). Targeted hepatocellular carcinoma proapoptotic BikDD gene therapy. Oncogene.

[27] Chou TC, Talalay P (1984). Quantitative analysis of dose-effect relationships: the combined effects of multiple drugs or enzyme inhibitors. Advances in enzyme regulation.

[28] Smith L, Watson MB, O'Kane SL, Drew PJ, Lind MJ, Cawkwell L (2006). The analysis of doxorubicin resistance in human breast cancer cells using antibody microarrays. Molecular cancer therapeutics.

[29] Minotti G, Menna P, Salvatorelli E, Cairo G, Gianni L (2004). Anthracyclines: molecular advances and pharmacologic developments in antitumor activity and cardiotoxicity. Pharmacological Reviews.

[30] Greene RF, Collins JM, Jenkins JF, Speyer JL, Myers CE (1983). Plasma pharmacokinetics of adriamycin and adriamycinol: implications for the design of *in vitro* experiments and treatment protocols. Cancer research.

[31] Finch RE, Bending MR, Lant AF (1979). Plasma levels of 5-fluorouracil after oral and intravenous administration in cancer patients. British Journal of Clinical Pharmacology.

[32] Findlay MP, Raynaud F, Cunningham D, Iveson A, Collins DJ, Leach MO (1996). Measurement of plasma 5-fluorouracil by high-performance liquid chromatography with comparison of results to tissue drug levels observed using *in vivo* 19F magnetic resonance spectroscopy in patients on a protracted venous infusion with or without interferon-alpha. Annals of oncology : official journal of the European Society for Medical Oncology/ESMO.

[33] Saif MW, Choma A, Salamone SJ, Chu E (2009). Pharmacokinetically guided dose adjustment of 5-fluorouracil: a rational approach to improving therapeutic outcomes. Journal of the National Cancer Institute.

[34] Shimazu T, Degenhardt K, Nur EKA, Zhang J, Yoshida T, Zhang Y, Mathew R, White E, Inouye M (2007). NBK/BIK antagonizes MCL-1 and BCL-XL and activates BAK-mediated apoptosis in response to protein synthesis inhibition. Genes & development.

[35] Li CH, Chen PY, Chang UM, Kan LS, Fang WH, Tsai KS, Lin SB (2005). Ganoderic acid X, a lanostanoid triterpene, inhibits topoisomerases and induces apoptosis of cancer cells. Life sciences.

[36] Scharovsky OG, Mainetti LE, Rozados VR (2009). Metronomic chemotherapy: changing the paradigm that more is better. Current Oncology.

[37] Shiraga E, Barichello JM, Ishida T, Kiwada H (2008). A metronomic schedule of cyclophosphamide combined with PEGylated liposomal doxorubicin has a highly antitumor effect in an experimental pulmonary metastatic mouse model. International Journal of Pharmaceutics.

[38] Gasparini G (2001). Metronomic scheduling: the future of chemotherapy?. The lancet oncology.

[39] Kamat AA, Kim TJ, Landen CN, Lu C, Han LY, Lin YG, Merritt WM, Thaker PH, Gershenson DM, Bischoff FZ, Heymach JV, Jaffe RB, Coleman RL, Sood AK (2007). Metronomic chemotherapy enhances the efficacy of antivascular therapy in ovarian cancer. Cancer research.

[40] Orlando L, Cardillo A, Rocca A, Balduzzi A, Ghisini R, Peruzzotti G, Goldhirsch A, D'Alessandro C, Cinieri S, Preda L, Colleoni M (2006). Prolonged clinical benefit with metronomic chemotherapy in patients with metastatic breast cancer. Anti-cancer drugs.

[41] Correale P, Cerretani D, Remondo C, Martellucci I, Marsili S, La Placa M, Sciandivasci A, Paolelli L, Pascucci A, Rossi M, Di Bisceglie M, Giorgi G, Gotti G, Francini G (2006). A novel metronomic chemotherapy regimen of weekly platinum and daily oral etoposide in high-risk non-small cell lung cancer patients. Oncology reports.

[42] Bocci G, Tuccori M, Emmenegger U, Liguori V, Falcone A, Kerbel RS, Del Tacca M (2005). Cyclophosphamide-methotrexate ‘metronomic’ chemotherapy for the palliative treatment of metastatic breast cancer. A comparative pharmacoeconomic evaluation. Annals of oncology : official journal of the European Society for Medical Oncology/ESMO.

[43] Wang S, Konorev EA, Kotamraju S, Joseph J, Kalivendi S, Kalyanaraman B (2004). Doxorubicin induces apoptosis in normal and tumor cells via distinctly different mechanisms. intermediacy of H(2)O(2)- and p53-dependent pathways. The Journal of biological chemistry.

[44] Xu Q, Leong J, Chua QY, Chi YT, Chow PK, Pack DW, Wang CH (2013). Combined modality doxorubicin-based chemotherapy and chitosan-mediated p53 gene therapy using double-walled microspheres for treatment of human hepatocellular carcinoma. Biomaterials.

[45] Panaretakis T, Pokrovskaja K, Shoshan MC, Grander D (2002). Activation of Bak, Bax, and BH3-only proteins in the apoptotic response to doxorubicin. The Journal of biological chemistry.

[46] Hur J, Bell DW, Dean KL, Coser KR, Hilario PC, Okimoto RA, Tobey EM, Smith SL, Isselbacher KJ, Shioda T (2006). Regulation of expression of BIK proapoptotic protein in human breast cancer cells: p53-dependent induction of BIK mRNA by fulvestrant and proteasomal degradation of BIK protein. Cancer research.

[47] Stravopodis DJ, Karkoulis PK, Konstantakou EG, Melachroinou S, Lampidonis AD, Anastasiou D, Kachrilas S, Messini-Nikolaki N, Papassideri IS, Aravantinos G, Margaritis LH, Voutsinas GE (2009). Grade-dependent effects on cell cycle progression and apoptosis in response to doxorubicin in human bladder cancer cell lines. International journal of oncology.

