# Supplementary material for: Targeted expression of BikDD combined with metronomic doxorubicin induces synergistic antitumor effect through Bax activation in hepatocellular carcinoma
Source: Oncotarget. 2015 Jun 17;6(27):23807–19. doi: 10.18632/oncotarget.4278 (PMC4695153; doi:10.18632/oncotarget.4278)
Supplement: Supplementary file 1 [file oncotarget-06-23807-s001.pdf]

SUPPLEMENTARY FIGURES AND TABLES

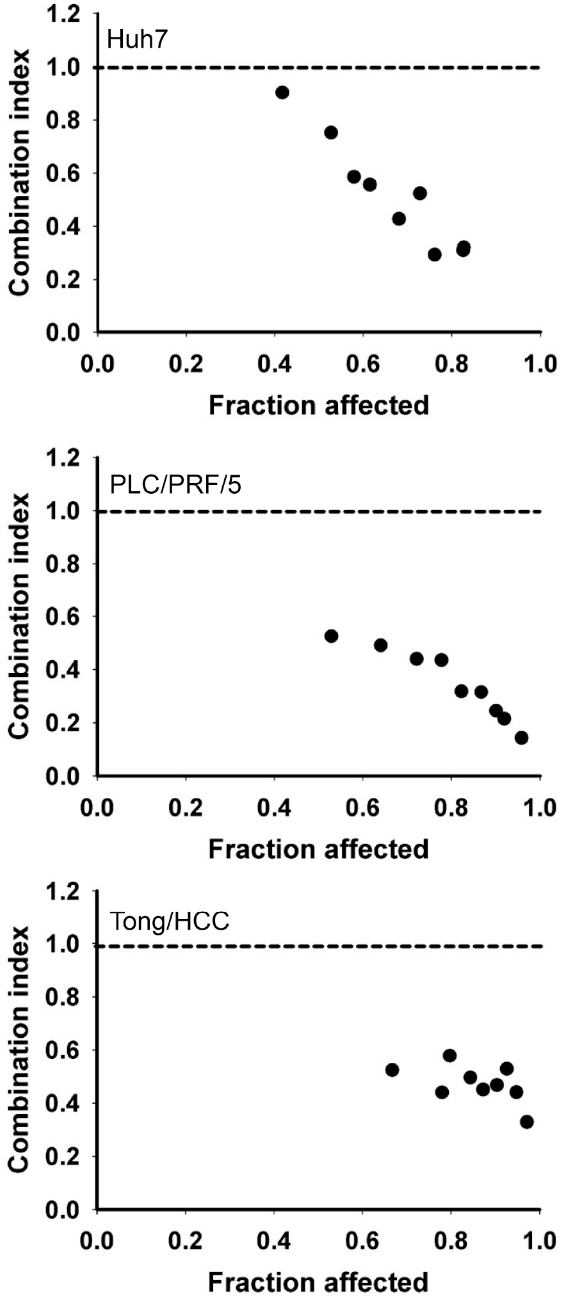

Supplementary Figure 1: Synergistic effects of eAFP-VISA-BikDD plus Dox against human HCC cell lines. Combination indices (CIs) of eAFP-VISA-BikDD combined with Dox versus the inhibition of cell survival (fraction affected; FA) were calculated by the CalcuSyn software. CI values > 1, = 1 and < 1 represent antagonism, additive effect and synergism, respectively. Top, Huh7; middle, PLC/PRF/5; and bottom, Tong/HCC cells.

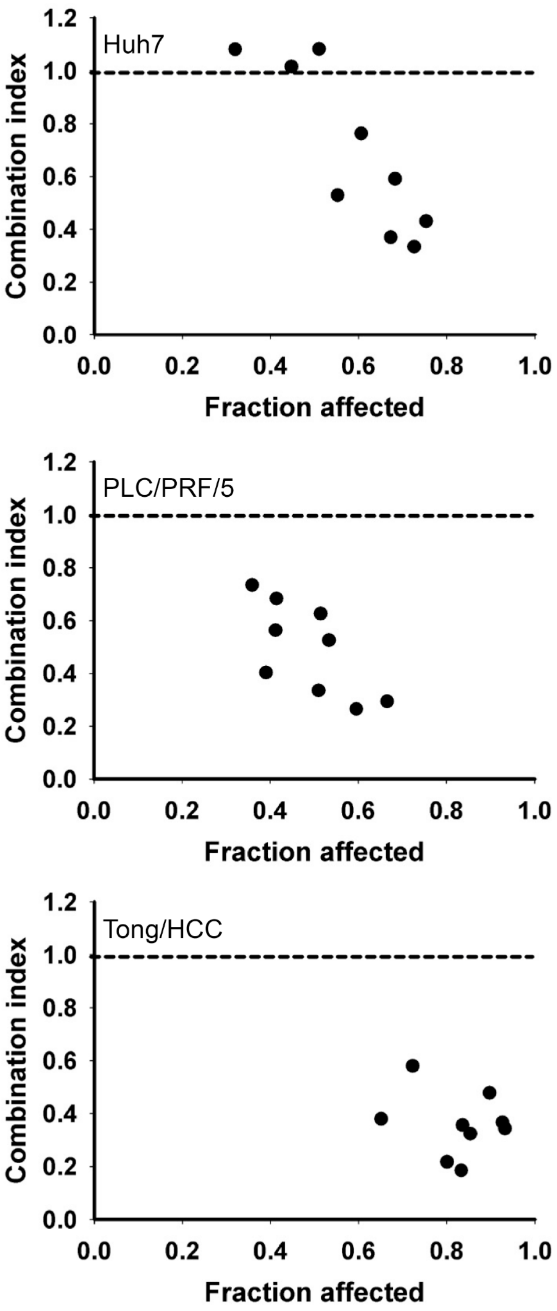

Supplementary Figure 2: Synergistic effects of eAFP-VISA-BikDD plus 5-FU against human HCC cell lines. Combination index (CI) plots of eAFP-VISA-BikDD plus 5-FU versus the inhibition of cell survival (fraction affected; FA) were calculated by the CalcuSyn software. CI values > 1, =1 and < 1 represent antagonism, additive effect or synergism, respectively. Top, Huh7; middle, PLC/PRF/5; and bottom, Tong/HCC cells.

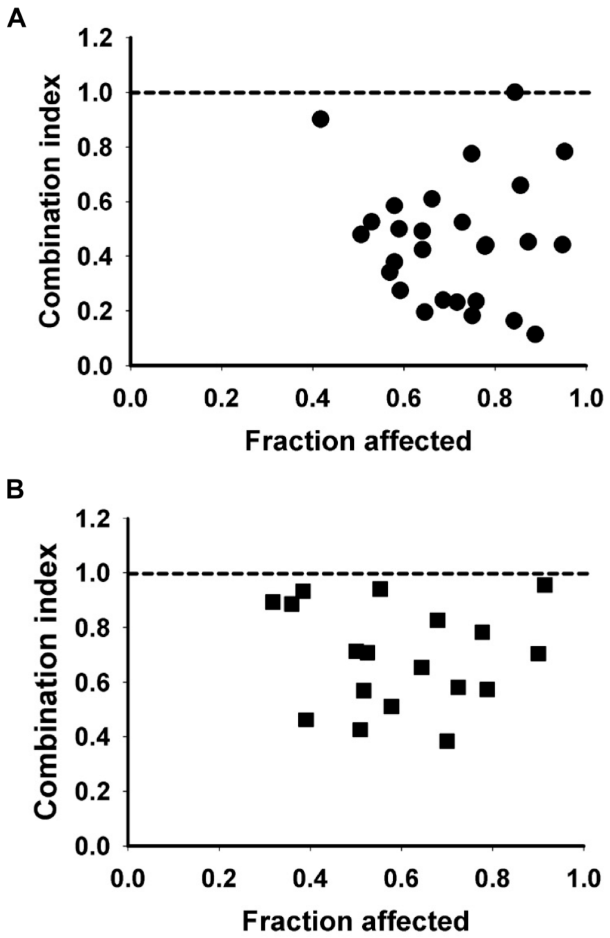

Supplementary Figure 3: Comparison of the killing effects of eAFP-VISA-BikDD/Dox versus eAFP-VISA-BikDD/5-FU under pathologically relevant concentration in HCC. Recalculation of the CI index from various concentration of eAFP-VISA-BikDD combined with clinically achievable doses of 0.1 µM Dox **A.** or 5 µM 5-FU **B.** in HCC cell lines from three independent experiments.

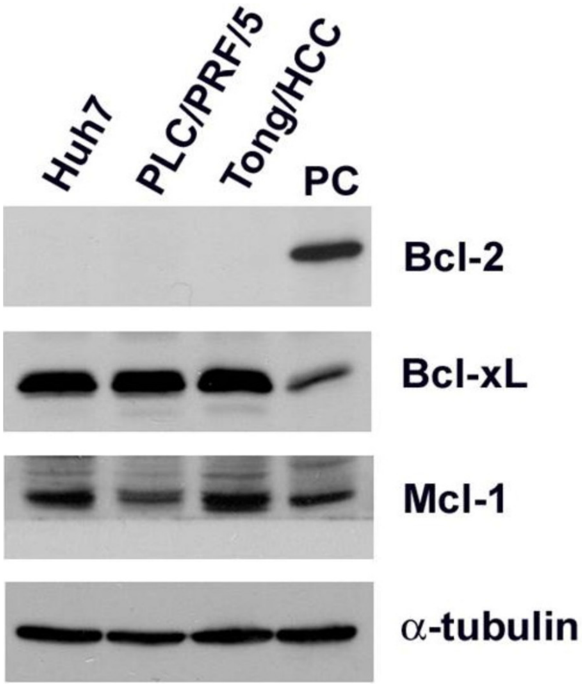

Supplementary Figure 4: Expression of anti-apoptotic proteins in HCC cell lines. Huh7, PLC/PRF/5 and Tong/HCC cell lysates were subjected to Western blot analysis with the indicated antibodies. Cell extract from Jurkat cells served as positive control (PC).

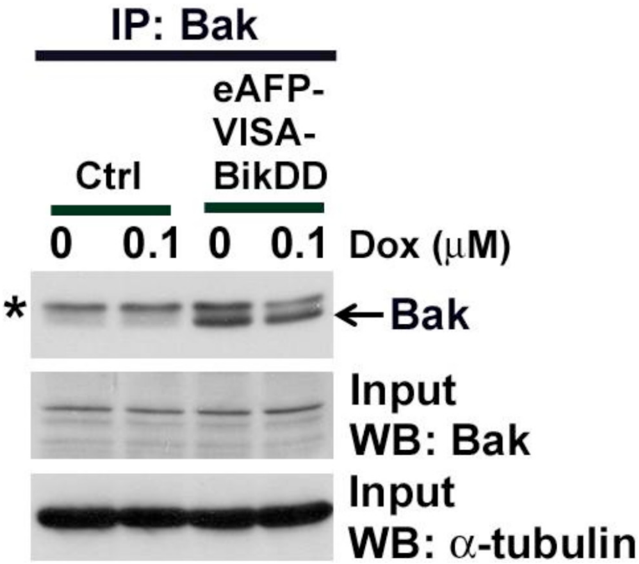

Supplementary Figure 5: Activated Bak levels in combination therapy. Huh7 cells were transfected with eAFP-VISA-BikDD for 4 hours. After transfection, the transfection mixture was replaced by DMEM complete medium containing Dox (0.1 μM) for additional 24 hours. Cell lysates were collected in 1% CHAPS lysis buffer and subjected to immunoprecipitation with anti-Bak antibody. Asterisk denotes the IgG light chain.

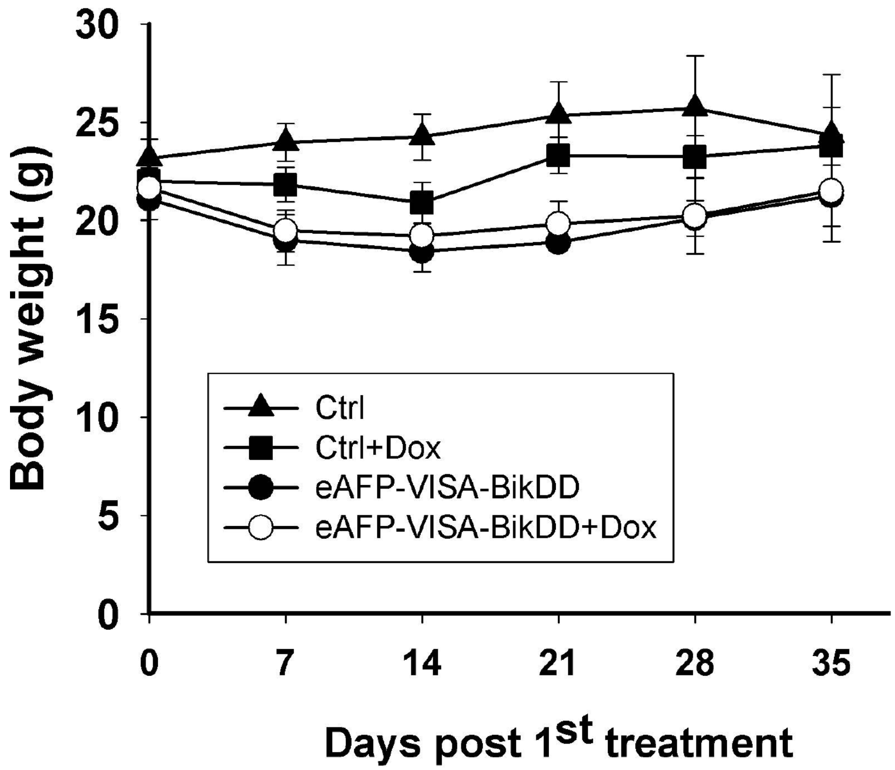

Supplementary Figure 6: Body weight of mice during the indicated treatment. Huh7 tumor-bearing SCID mice were administered the indicated treatment as described in Materials and Methods. Body weight was monitored and recorded weekly. Values are presented as mean ±SD.

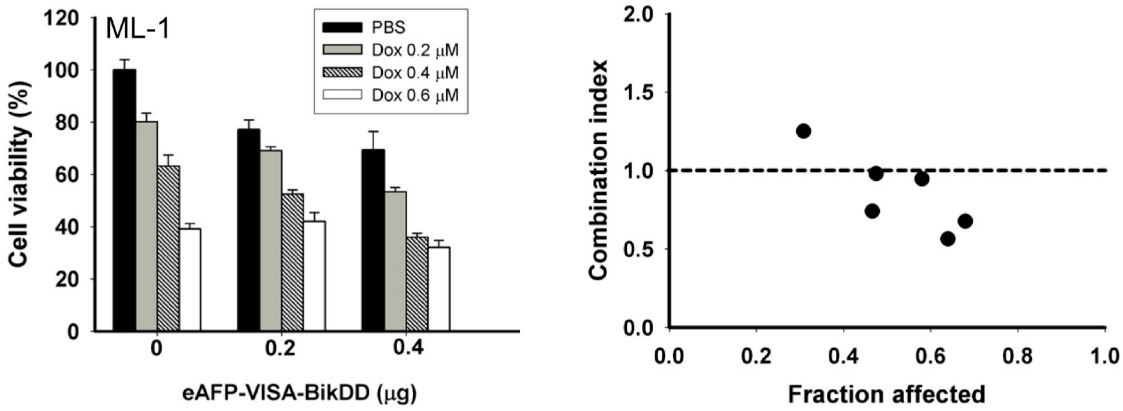

Supplementary Figure 7: Synergistic effects of combination therapy of eAFP-VISA-BikDD and Dox in ML-1 cells *in vitro*. ML-1 cells were transfected with indicated concentrations of eAFP-VISA-BikDD for 4 hours. Transfection mixtures were then replaced by fresh DMEM complete medium containing various concentrations of Dox for an additional 72 hours. Cytotoxic effects were evaluated by sulforhodamine B (SRB) assay. Relative cell viability was normalized to untreated cells (set as 100%). Data represent mean ±SD. Combination indices (CIS) were calculated by the CalcuSyn software. CI values > 1, =1 and < 1 represent antagonism, additive effect, and synergism, respectively.

Supplementary Table 1: Combination therapy enhances apoptosis in HCC cells *in vitro*

Treatment Groups	Huh7	PLC/PRF/5	Tong/HCC
Control (Ctrl)	4.88 ± 0.28	0.32 ± 0.08	4.46 ± 0.01
Dox (0.1 µM)	14.3 ± 0.01	6.5 ± 0.81	14.56 ± 0.23
eAFP-VISA-BikDD	30.44 ± 1.01	23.84 ± 2.4	27.7 ± 1.01
eAFP-VISA-BikDD/Dox (0.1 µM)	42.37 ± 2.34	34.86 ± 3.82	34.93 ± 0.94

Data represent mean ±SD from 3 independent experiments. Values shown are percentage of Sub-G1 cells.

Supplementary Table 2: Combination therapy inhibits metastasis of HCC

Treatment Groups	Mean number of pulmonary metastases
Control (Ctrl)	47.2 ± 8.7
Dox (0.5 mg/kg)	64.4 ± 13.5
eAFP-VISA-BikDD	2.2 ± 1.0*
eAFP-VISA-BikDD+ Dox (0.5 mg/kg)	1.2 ± 1.2*

Data represent mean ±SEM. $N = 5$. *$P < 0.05$.

Supplementary Table 3: Molar ratios for the eAFP-VISA-BikDD and Dox combination in HCC cells

Cell line	eAFP-VISA-BikDD (μg)	Dox (μM)	Combination index	Molar ratio (eAFP-VISA-BikDD:Dox)
Huh7	0.05	0.1	0.9	1:1
	0.05	0.25	0.75	1:2
	0.05	0.4	0.56	1:4
	0.1	0.1	0.59	2:1
	0.1	0.25	0.43	1:1
	0.1	0.4	0.29	1:2
	0.2	0.1	0.52	4:1
	0.2	0.25	0.31	2:1
	0.2	0.4	0.32	1:1
PLC/PRF/5	0.1	0.1	0.53	1:2
	0.1	0.25	0.44	1:4
	0.1	0.5	0.32	1:8
	0.2	0.1	0.49	1:1
	0.2	0.25	0.32	1:2
	0.2	0.5	0.22	1:4
	0.4	0.1	0.44	2:1
	0.4	0.25	0.25	1:1
	0.4	0.5	0.14	1:2
Tong/HCC	0.1	0.05	0.52	2:1
	0.1	0.1	0.44	1:1
	0.1	0.25	0.50	1:2
	0.2	0.05	0.58	4:1
	0.2	0.1	0.45	2:1
	0.2	0.25	0.47	1:1
	0.4	0.05	0.53	8:1
	0.4	0.1	0.44	4:1
	0.4	0.25	0.33	2:1
